# Atypical Manifestation of COVID-19-Induced Myocarditis

**DOI:** 10.7759/cureus.8685

**Published:** 2020-06-18

**Authors:** Mahin Rehman, Amlish Gondal, Najeeb U Rehman

**Affiliations:** 1 Internal Medicine, Guthrie Clinic, Robert Packer Hospital, Sayre, USA; 2 Cardiology, Guthrie Clinic, Robert Packer Hospital, Sayre, USA

**Keywords:** pandemic, covid-19 pandemic, cardiology, st-elevation myocardial infarction (stemi), covid-19, sars-cov-2, covid-induced myocarditis, myocarditis, world pandemic, viral myocarditis

## Abstract

We present a case of a 39-year-old male who presented with chest pain without fever or respiratory symptoms. Troponins were elevated and electrocardiogram (ECG) was inconclusive for ST-elevation myocardial infarction (STEMI). Angiography revealed normal coronaries and the patient was found to be coronavirus disease 2019 (COVID-19) positive; he was diagnosed with COVID-19 myocarditis. With the global pandemic, more cases are emerging regarding myocardial injury induced by severe acute respiratory syndrome coronavirus 2 (SARS-CoV-2) virus. Although COVID-19 manifests primarily as respiratory disease, few cases of cardiac injury without respiratory involvement or febrile illness have been reported. This case illustrates that COVID-19 can present atypically and affect an isolated non-respiratory organ system.

## Introduction

Coronavirus disease 2019 (COVID-19) (severe acute respiratory syndrome coronavirus 2 [SARS-CoV-2]) not only affects the respiratory system but can involve dysfunction of other organ systems as well. There have been reports of myocardial injury occurring in patients who have tested positive for COVID-19 with evidence of troponin leak and elevation, and these patients may initially present with a pseudoinfarct pattern [1]. Additionally, fever, cough, and respiratory distress were highly common symptoms noted in individuals who had myocardial injury associated with COVID-19 [1].

The SARS-CoV-2 virus is not known to be cardiotropic like other viruses such as the coxsackie virus. The virus attacks by attaching to the angiotensin-converting-enzyme-2 (ACE2) receptor which is located in the lungs but also found on the heart and vessels; a cytokine release storm occurs due to an imbalance of T-cell activation with improper release of cytokines such as interleukin (IL)-6, IL-17 and others, leading to the possibility of significant cellular damage [2]. Additionally, this severe immune system response may also result in plaque instability and ultimately lead to the development of an acute coronary syndrome (ACS) [2].

Currently, there have been no reports confirming SARS-CoV-2-induced viral myocarditis via histologic and viral genomic analysis through polymerase chain reaction (PCR); molecular proof and evidence are still needed via biopsy identifying the SARS-CoV-2 genome within myocytes. Thus, these reports of COVID-19 myocarditis are all clinical diagnoses. There was a case report that described a patient with COVID-19 with regional wall motion abnormalities who had a biopsy consistent with lymphocytic myocarditis but histopathological and viral genomic polymerase chain reaction (PCR) analysis of the biopsy did not reveal the SARS-CoV-2 viral genome to be present within the myocytes [3]. One case series of 18 patients with COVID-19 associated ST-segment elevation on an electrocardiogram (ECG); myocardial infarction was diagnosed in eight of these individuals with the remaining 10 being diagnosed with noncoronary myocardial injury [4].

Currently, there have been no COVID-19 related reports of isolated myocardial injury without febrile illness and respiratory involvement.

## Case presentation

History of presentation and past medical history

A 39-year-old Kenyan male with no past medical history presented with midsternal chest pain. He had been residing within the USA for the last year and working as a long-distance truck driver. The patient described the pain as a heaviness which sometimes felt sharp and lasted several hours long. He had been experiencing this pain for two days and the pain usually started in the morning and worsened with exertion. On the day of presentation, he rated the pain 9 out of 10 on the severity scale and denied any symptoms of shortness of breath or pleuritic type chest discomfort associated with respiration. He was administered aspirin and sublingual nitroglycerin; the nitroglycerin did not relieve his chest pain and his presentation was worrisome for an ST-elevation myocardial infarction (STEMI). The patient was completely afebrile and vital signs were stable. Physical examination only revealed some elicited pain with range of motion of his left arm. He denied any alcohol use, tobacco use, or illicit drug use and denied any family history of cardiac disease. 

Investigations

**Figure 1:**
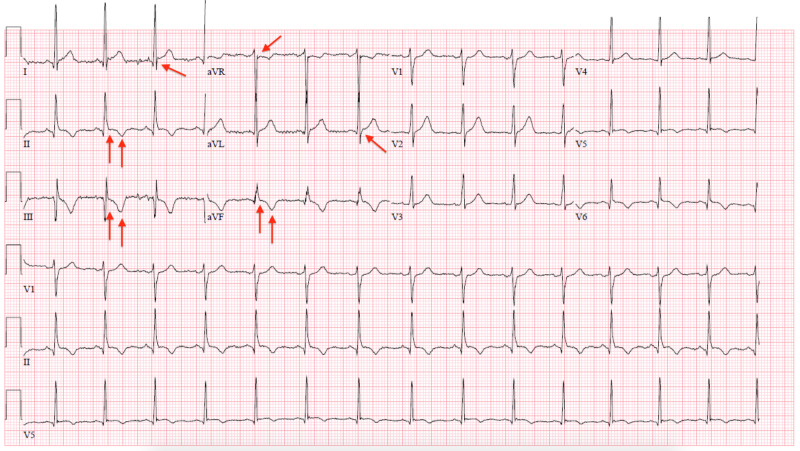
Electrocardiograph (ECG) of the patient with coronavirus disease 2019 (COVID-19) The red arrows in this ECG show 1 to 2 mm ST elevations in lead I and aVL, ST depression in aVR, mild J-point elevation and T-wave inversion in leads II, III and aVF.

**Figure 2:**
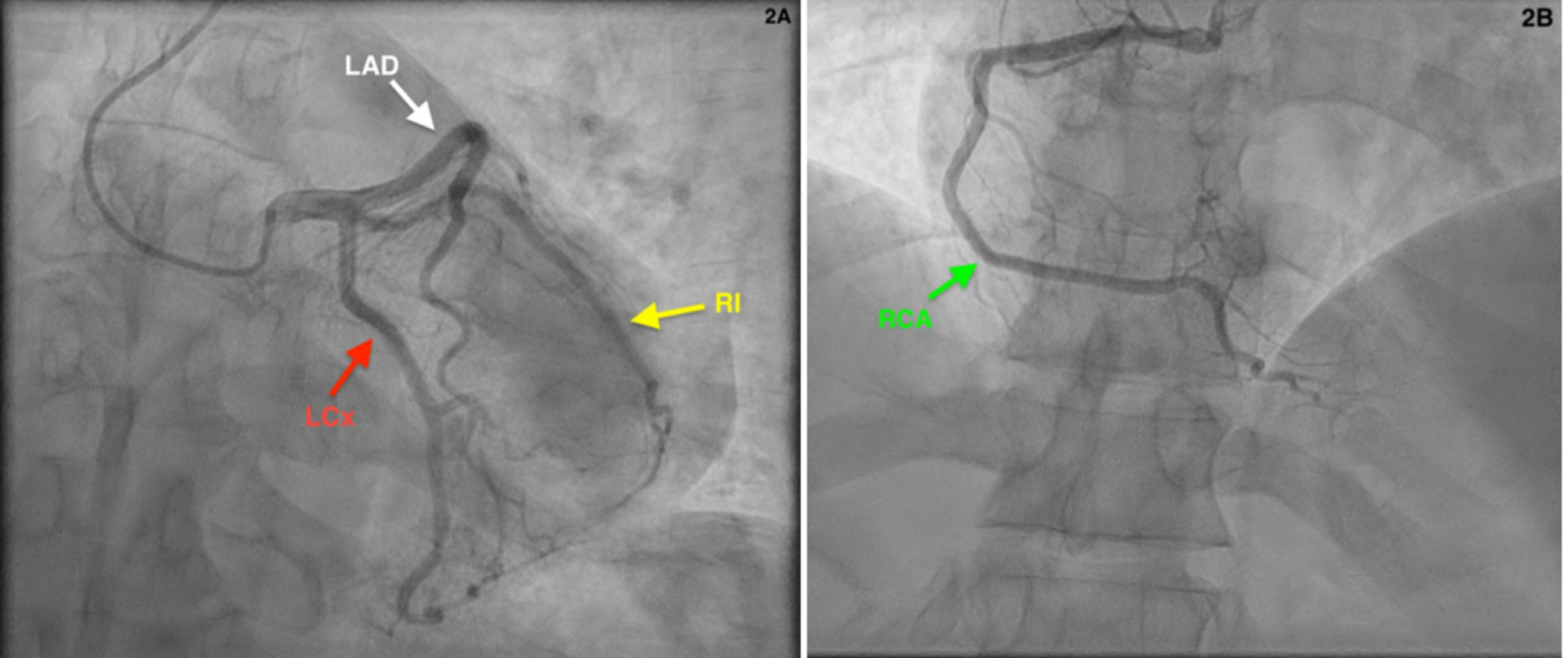
Coronary angiography showing normal coronary arteries A: Red arrow showing patent left circumflex artery (LCx). White arrow showing patent left anterior descending artery (LAD). Yellow arrow showing patent ramus intermidius artery (RI). B: Green arrow showing patent right coronary artery (RCA).

His ECG showed 1 to 2 mm ST elevations in lead I and aVL, ST depression in aVR, mild J-point elevation, and T-wave inversion in leads II, III and aVF (Figure 1). His ECG was not entirely convincing of STEMI and his troponins were found to be elevated at 5.97 ng/mL and eventually peaked to 6.24 ng/mL. He was taken urgently for cardiac catheterization and it revealed completely normal coronaries so no intervention was performed (Figures 2A-2B). There was no evidence of myocardial bridging either. 

Given his profession of being a long-distance truck driver and traveling, he was tested via reverse transcription polymerase chain reaction (RT-PCR) for COVID-19 (SARS-CoV-2 virus) and was found to be positive. Subsequently, a CT scan was done to rule out pulmonary embolism as the cause of his chest pain since SARS-CoV-2 virus induces a hypercoagulable and thrombotic state in individuals [5]. CT scan was negative for any pulmonary embolism and revealed clear lungs without any typical respiratory characteristics of COVID-19. This was followed up with a transthoracic echocardiography (TTE) and it revealed no wall motion abnormalities and a completely normal ejection fraction at 55%-60% and no evidence of pericarditis or pericardial effusion. 

The patient's blood work was consistent with biomarkers typically identified with COVID-19 (SARS-CoV-2): his d-dimer was elevated at 0.96 mcg/mL, erythrocyte sedimentation rate (ESR) elevated at 44 mm/hr, lactate dehydrogenase (LDH) elevated at 926 units/L, procalcitonin was negative at 0.04 ng/mL, C-reactive protein (CRP) elevated at 3.3 mg/dL, creatine phosphokinase (CPK) elevated at 366 units/L, amino-terminal pro-brain natriuretic peptide (NT-proBNP) elevated at 379 pg/mL, and mild to moderate liver enzyme elevation with aspartate aminotransferase (AST) elevated at 89 units/L and alanine aminotransferase (ALT) elevated at 152 units/L [6]. COVID-19 is commonly associated with elevated ferritin levels since ferritin is an acute phase reactant but interestingly, in our patient, the ferritin was within normal limits at 243 ng/mL [6].

Differential diagnosis

Initially, STEMI was at the top of the differential given that he had an equivocal ECG and worsening chest pain. With cardiac catheterization revealing normal coronary arteries and no lesion inducing stenosis, myocarditis became the top differential given that he had evidence of myocardial injury with the troponin leak. Cardiac catheterization also ruled out any concern for myocardial bridging as the source of his chest pain. 

Management and follow-up

The patient was managed conservatively during his hospitalization. Interestingly, after his cardiac catheterization, his chest pain resolved and the pain was well-controlled with acetaminophen alone. He was not on any medications prior to admission and was discharged in a stable condition with acetaminophen and non-steroidal anti-inflammatory drugs (NSAIDs) were avoided for the concern of exacerbating myocardial inflammation due to COVID-19. The patient is scheduled for follow-up with repeat serial TTE to ensure that he is not developing heart failure with reduced ejection fraction or wall motion abnormalities.

## Discussion

With this report, we aim to highlight an atypical presentation of COVID-19 (SARS-CoV-2)-induced myocarditis as this patient was completely afebrile and had no respiratory symptoms, both of which are typical characteristics. Current consensus around COVID-19-induced myocardial injury is to maintain conservative management especially in those without suspected acute coronary syndrome (ACS) who have mild troponin elevation, as in our young patient. Given that he is young, was recovering well, and did not present with acute heart failure, he was conservatively managed and carefully monitored without requiring further cardiac imaging such as magnetic resonance imaging (MRI) as it would not have altered our management [7].

With regards to not using NSAIDs in our patient, we erred on the side of caution. There was some concern about NSAIDs possibly exacerbating the COVID-19 disease course early on in the pandemic but the World Health Organization (WHO) released a publication addressing this and concluding that there is no evidence of severe adverse effects in COVID-19 patients treated with NSAIDs [8]. Thus, for future COVID-19 patients, NSAIDs may be utilized should they be warranted.

Another major factor to consider with this case is the exposure of the cardiovascular team to SARS-CoV-2 virus without proper personal protective equipment (PPE) and appropriate precautions. He was taken urgently to the catheterization lab as there was concern for STEMI and at that point, the disease was quite low on the differential since the patient did not have typical signs and symptoms of COVID-19. The European Society of Cardiology (ESC) has released guidelines on how cardiovascular healthcare personnel can protect themselves [2]. For percutaneous coronary intervention (PCI), they recommend that for suspected and probable COVID-19 positive patients, a disposable surgical cap, an N95 respirator, a disposable gown, disposable gloves, and eye goggles or face shield be utilized [2]. Again, our patient had no fever or respiratory symptoms of the disease, so it was very low on our differential and ultimately thorough protection was not worn. The cardiovascular team was tested for the virus as well and monitored for symptoms. The cardiac catheterization lab and equipment was thoroughly sanitized and disinfected; while the catheterization lab is not in use, ultraviolet light is left on for continuous disinfection. 

Again, there have been no reports confirming the presence of SARS-CoV-2 viral genome in myocytes via endomyocardial biopsy showing lymphocytic infiltrate; once biopsy-proven invasion of myocardial cells by SARS-CoV-2 has been reported, then a new cardiotropic virus inducing myocarditis would be added to the literature. 

## Conclusions

COVID-19-induced myocardial injury can present as a STEMI or non-STEMI (given the evidence of troponin leak) and without concurrent febrile illness or respiratory symptoms of the disease. Conservative management with pain control is recommended with serial TTEs on follow-up. NSAIDs may be utilized, should it be warranted as per the WHO, as there is no significant data indicating they exacerbate the disease course or induce harm to the patients.
